# Supplementary material for: Common garden experiment reveals altered nutritional values and DNA methylation profiles in micropropagated three elite Ghanaian sweet potato genotypes
Source: PLoS One. 2019 Apr 26;14(4):e0208214. doi: 10.1371/journal.pone.0208214 (PMC6485893; doi:10.1371/journal.pone.0208214)
Supplement: S1 Fig — Micropropagated (shown in orange border) and field-maintained plants (black bordered) planted in a common garden in a Randomized Complete Block Design with three replicates. Each block consisted of three plots: Bo (Bohye), Og (Ogyefo), and Ot (Otoo). Two Ridges (4.5 m long), spaced at 1 m, were made on each plot and fourteen vine cuttings of about 30 cm were planted on each ridge, with an interval of 30 cm between plants. The blocks were bordered with guard rows to reduce the effects of biotic and abiotic factors on the edge rows. (DOCX) [file pone.0208214.s001.docx]

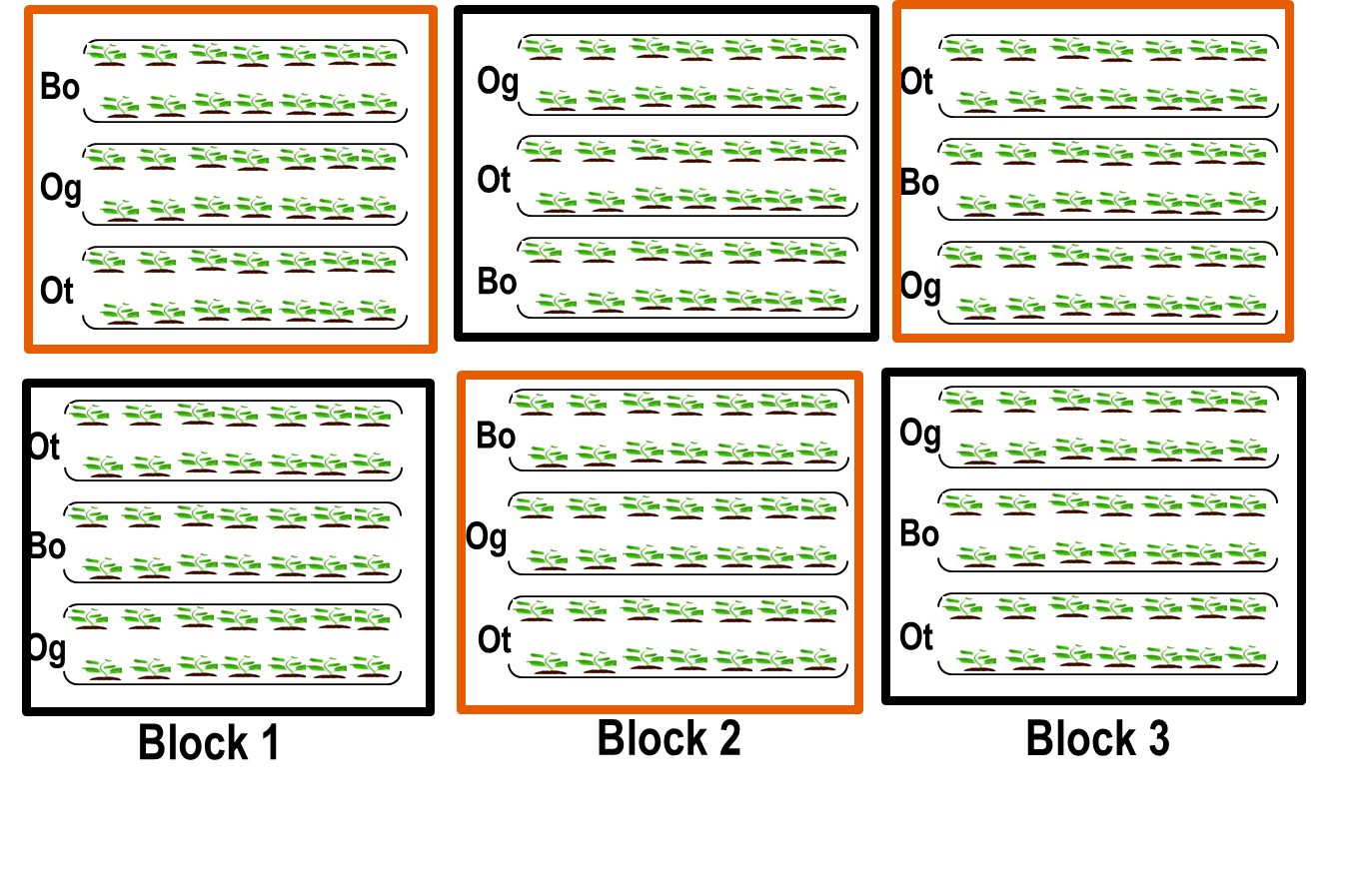


**Figure S1.** Experimental layout. Micropropagated (Blocks with orange border) and field-maintained plants (Locks with black border) planted in a common garden in a Randomized Complete Block Design with three replicates. Each block consisted of three plots each planted with a different sweet potato genotype: Bo (Bohye), Og (Ogyefo), and Ot (Otoo). Two Ridges (4.5 m long), spaced at 1 m, were made on each plot and fourteen vine cuttings of about 30 cm were planted on each ridge, with an interval of 30 cm between plants. The blocks were bordered with guard rows to reduce the effects of biotic and abiotic factors on the edge rows.
