# Supplementary figures and images for: Common garden experiment reveals altered nutritional values and DNA methylation profiles in micropropagated three elite Ghanaian sweet potato genotypes
Source: PLoS One. 2019 Apr 26;14(4):e0208214. doi: 10.1371/journal.pone.0208214 (PMC6485893; doi:10.1371/journal.pone.0208214)

# **
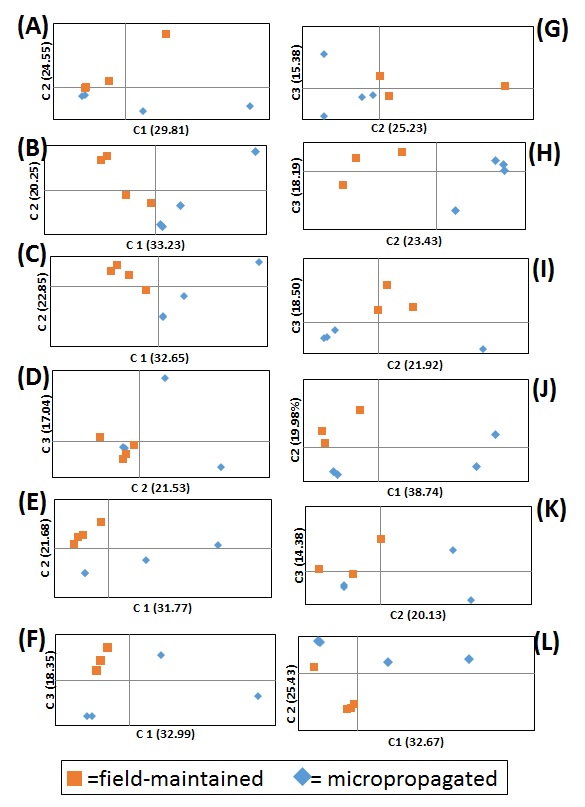
**

**Figure S2.** PCoA results of the 12 primer combinations used for MSAP pilot studies.

Supplement: S2 Fig — (DOCX) [file pone.0208214.s002.docx]
