# Supplementary material for: Common garden experiment reveals altered nutritional values and DNA methylation profiles in micropropagated three elite Ghanaian sweet potato genotypes
Source: PLoS One. 2019 Apr 26;14(4):e0208214. doi: 10.1371/journal.pone.0208214 (PMC6485893; doi:10.1371/journal.pone.0208214)
Supplement: S3 Fig — A) PCoA generated using GenAlex v6.5 from MSAP profiles from micropropagated (empty symbols) and field-maintained (full symbols) plants from genotypes of Bohye (green), Ogyefo (red), and Otoo (blue) (n = 24). MSAP profiles were amplified from genomic DNA restricted using HpaII (circles) or MspI (squares) and amplified using primer combinations E and I (Results were calculated using loci from both primer combinations together). B) PCA generated by msapR 3.3.1 using MSAP profiles as above. Label on centroids indicate genotype (i.e. Bohye (Bo), Ogyefo (Og), and Otoo (Ot)) and type of propagule (i.e. Field maintained (FM) and micropropagated (VF)). (DOCX) [file pone.0208214.s003.docx]

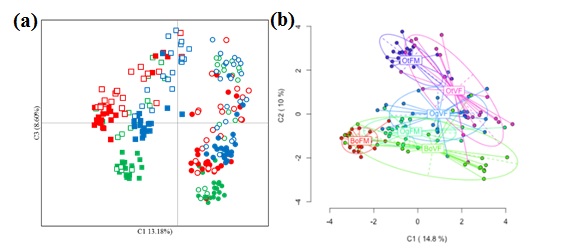


**Figure S3.** **Analysis of molecular somaclonal variation induced by micropropagation of sweet potato. A)** PCoA generated using GenAlex v6.5 from MSAP profiles from micropropagated (empty symbols) and field-maintained (full symbols) plants from genotypes of Bohye (green), Ogyefo (red), and Otoo (blue) (n = 24). MSAP profiles were amplified from genomic DNA restricted using *Hpa*II (circles) or *Msp*I (squares) and amplified using primer combinations E and I (Results were calculated using loci from both primer combinations together). **B)** PCA generated by *msap*R 3.3.1 using MSAP profiles as above. Label on centroids indicate genotype (i.e. Bohye (Bo), Ogyefo (Og), and Otoo (Ot)) and type of propagule (i.e. Field maintained (FM) and micropropagated (VF)).
