# Supplementary material for: Common garden experiment reveals altered nutritional values and DNA methylation profiles in micropropagated three elite Ghanaian sweet potato genotypes
Source: PLoS One. 2019 Apr 26;14(4):e0208214. doi: 10.1371/journal.pone.0208214 (PMC6485893; doi:10.1371/journal.pone.0208214)
Supplement: S4 Fig — PCA generated by msapR 3.3.1 from MSAP profiles from micropropagated (blue symbols) and field-maintained (red symbols) plants from genotypes of Bohye (a and d), Ogyefo (b and e), and Otoo (c and f) (n = 24). MSAP profiles were amplified from genomic DNA restricted using HpaII and MspI (profiles combined for analysis) and amplified using primer combinations E (a-c) and I (d-f). Epigenetic variability was calculated using Methylation Sensitive Loci (a1-f1) and genetic variability using Non-Methylated Loci (a2-f2). Plants were grown on a Randomized Complete Block Design with three replicated blocks and 24 plants/block/genotype/propagation system. (DOCX) [file pone.0208214.s004.docx]

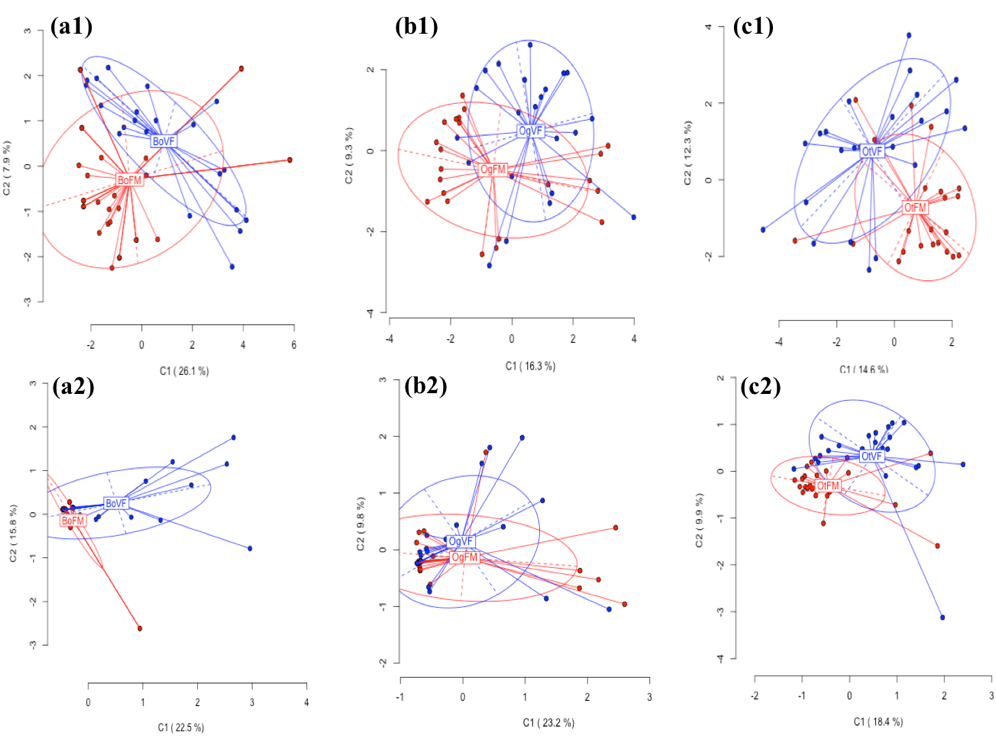

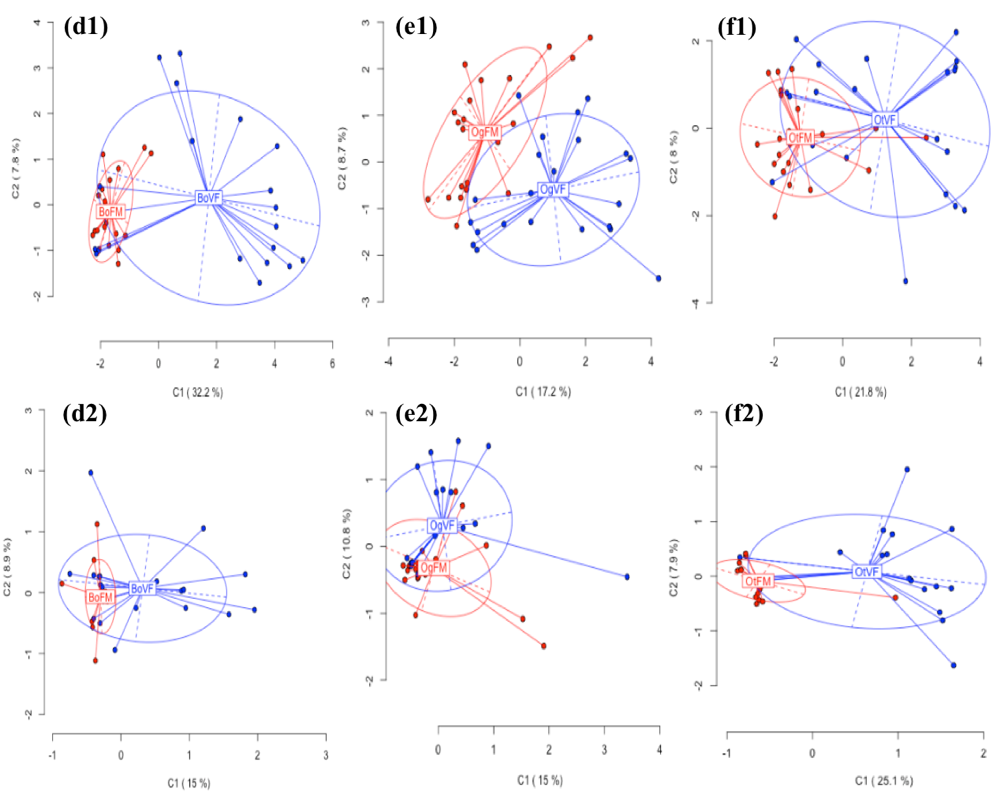


**Figure S4.** **Analysis of epigenetic and genetic variability induced by micropropagation in three sweet potato genotypes**. PCA generated by *msap*R 3.3.1 from MSAP profiles from micropropagated (blue symbols) and field-maintained (red symbols) plants from genotypes of Bohye (a and d), Ogyefo (b and e), and Otoo (c and f) (n=24). MSAP profiles were amplified from genomic DNA restricted using *Hpa*II and *Msp*I (profiles combined for analysis) and amplified using primer combinations E (a-c) and I (d-f). Epigenetic variability was calculated using Methylation Sensitive Loci (a1-f1) and genetic variability using Non-Methylated Loci (a2-f2). Plants were grown on a Randomized Complete Block Design with three replicated blocks and 24 plants/block/genotype/propagation system.
