# Supplementary material for: Common garden experiment reveals altered nutritional values and DNA methylation profiles in micropropagated three elite Ghanaian sweet potato genotypes
Source: PLoS One. 2019 Apr 26;14(4):e0208214. doi: 10.1371/journal.pone.0208214 (PMC6485893; doi:10.1371/journal.pone.0208214)
Supplement: S1 Table — (DOCX) [file pone.0208214.s005.docx]

**Table S1.** Identity, origin, year of release, and preferred ecology of the three sweet potato genotypes (Bohye, Ogyefo, and Otoo) used in the study.

| Name of Genotype | CRI- Otoo | CRI-Ogyefo | CRI-Bohye |
| --- | --- | --- | --- |
| Pedigree Line | Mogamba | Not known | 199062.1 |
| National Code | GH/Ib/ 005/15 | GH/Ib/ 007/15 | GH/Ib/ 010/15 |
| Origin/ Source | CIP | CIP | CIP |
| Year of Release | 2005 | 2005 | 2012 |
| Year of initiation in tissue culture | 2013 | 2013 | 2012 |
| Date certified as virus-free | 04/2015 | 04/2015 | 05/2014 |
