# Supplementary material for: Common garden experiment reveals altered nutritional values and DNA methylation profiles in micropropagated three elite Ghanaian sweet potato genotypes
Source: PLoS One. 2019 Apr 26;14(4):e0208214. doi: 10.1371/journal.pone.0208214 (PMC6485893; doi:10.1371/journal.pone.0208214)
Supplement: S2 Table — (DOCX) [file pone.0208214.s006.docx]

**Table S2** Scale of reference (1-9) and definition of scores for virus incidence, foliar and root morphological descriptors.

| Character score | Definition of score |
| --- | --- |
| Virus scoring | 1=free 2=unsure 3=5% 4=15% 5=33% 6=66% 7=99% 8=66-100% 9=all viral |
| Immature foliage colour | 1=Yellow-green 2=Green 3=Green with purple edge 4=Greyish green (due to heavy pubescence) 5=Green with purple veins on upper surface 6=Slightly purple 7=Mostly purple 8=Green upper, purple lower 9=Purple both surfaces |
| Mature foliage colour | 1=Yellow-green 2=Green 3=Green with purple edge 4=Greyish green (due to heavy pubescence) 5=Green with purple veins on upper surface 6=Slightly purple 7=Mostly purple 8=Green upper, purple lower 9=Purple both surfaces |
| Abaxial leaf vein pigmentation (ALVP) | 1 =Yellow 2=Green 3= Purple spot at base of main rib 4 =Purple spots in several veins 5 =Main rib partially purple 6= Main rib mostly or totally purple 7= All veins partially purple 8= All veins mostly or totally purple 9= Lower surface and veins totally purple |
| Petiole pigmentation (PP) | 1= Green 2= Green with purple near stem 3= Green with purple near leaf 4= Green with purple at both ends 5= Green with purple spots throughout petiole 6= Green with purple stripes 7 Purple with green near leaf 8= Some, petioles purple, others green 9= Totally or mostly purple |
| Predominant vine colour (PVC) | 1= Green 3 =Green with few purple spots 4= Green with many purple spots 5= Green with many dark purple spots 6= Mostly purple7 =Mostly dark purple 8 =Totally purple 9 =Totally dark purple |
| Secondary vine colour (SVC) | Secondary vine colour 0= Absent 1= Green base 2= Green tip 3= Green nodes 4 =Purple base 5 =Purple tip 6= Purple nodes Vine tip pubescence 0= None 3 =Sparse 5= Moderate 7 =Heavy 9= Very heavy |
| Plant type (PT) | Determined by the length of the main vines 3 =Erect (<75 cm) 5 =Semi-compact (75 – 150 cm) 7= Spreading (151 – 250 cm) 9= Extremely spreading (>250 cm) |
| Predominant Skin colour | Predominant Skin colour 1= White 2= Cream 3= Yellow 4= Orange 5= Brownish orange 6= Pink 7= Red 8= Purple red 9= Dark purple |
| Storage root shape | 1= Round 2= Round elliptic 3= Elliptic 4 =Obovate 5= Ovate 6 =Oblong 7= Long oblong 8=Long elliptic 9 =Long irregular or curved. |
| Storage root flesh colour | 1=White 2=Pale Cream 3=Dark cream 4=Pale Yellow 5=Dark Yellow 6=Pale Orange 7=Intermediate Orange 8=Dark orange Pigmented with anthocyanin |
