# Supplementary material for: Common garden experiment reveals altered nutritional values and DNA methylation profiles in micropropagated three elite Ghanaian sweet potato genotypes
Source: PLoS One. 2019 Apr 26;14(4):e0208214. doi: 10.1371/journal.pone.0208214 (PMC6485893; doi:10.1371/journal.pone.0208214)
Supplement: S3 Table — Primer selective bases are highlighted in bold. (DOCX) [file pone.0208214.s007.docx]

**Table S3.** Oligonucleotides used during Methylation Sensitive Amplified Polymorphisms protocol with their sequences and function.

| **Primer name** | **Function** | **Sequence (5’-3’)** |
| --- | --- | --- |
| Ad. *Hpa*II/*Msp*I | Reverse Adaptor | CGCTCAGGACTCAT |
| Ad. *Hpa*II/*Msp*I | Forward adaptor | GACGATGAGTCCTGAG |
| Ad. *EcoR*I | Reverse adaptor | AATTGGTACGCAGTCTAC |
| *Ad. EcoR*I | Forward adaptor | CTCGTAGACTGCGTACC |
| Pre-*Eco*RI | Preselective primer | GACTGCGTACCAATTC**A** |
| Pre-*Hpa*II/*Msp*I | Preselective primer | GATGAGTCCTGAGCGG**C** |
| *EcoR*I 3.1 | Selective primer | GACTGCGTACCAATTC**AAA** |
| *EcoR*I 3.2 | Selective primer | GACTGCGTACCAATTC**AAC** |
| *EcoR*I 3.3 | Selective primer | GACTGCGTACCAATTC**AAG** |
| *EcoR*I3.10 | Selective primer | GACTGCGTACCAATTC**AGC** |
| *EcoR*I 3.13 | Selective primer | GACTGCGTACCAATTC**ATA** |
| *EcoR*I 3.15 | Selective primer | GACTGCGTACCAATTC**ATG** |
| *HpaII/MspI* 3.1 | Selective primer | [6FAM]GATGAGTCCTGAGCGG**CAA** |
| *HpaII/MspI* 3.5 | Selective primer | [6FAM]GATGAGTCCTGAGCGG**CCA** |

Primer selective bases are highlighted in bold.
