# Supplementary material for: Common garden experiment reveals altered nutritional values and DNA methylation profiles in micropropagated three elite Ghanaian sweet potato genotypes
Source: PLoS One. 2019 Apr 26;14(4):e0208214. doi: 10.1371/journal.pone.0208214 (PMC6485893; doi:10.1371/journal.pone.0208214)
Supplement: S4 Table — Primers selected are indicated with an asterisk. The number of alleles (# of loci), percentage reproducibility of alleles (% Rep), and number of differential alleles (# diff. alleles) are displayed. (DOCX) [file pone.0208214.s008.docx]

**Table S4.** Results of **s**elective primer combinations for MSAP pilot study.

| Primer  Combination | *HpaII/ MspI* | *EcoR*I | # of loci | % Rep. | # diff. alleles |
| --- | --- | --- | --- | --- | --- |
| A | *HpaII/MspI* 3.5 | *EcoR*I 3.1 | 167 | 97.60 | 5 |
| B | *HpaII/MspI* 3.5 | *EcoR*I 3.2 | 165 | 92.00 | 10 |
| C | *HpaII/MspI* 3.5 | *EcoR*I 3.3 | 167 | 93.00 | 12 |
| D | *HpaII/MspI* 3.5 | *EcoR*I 3.15 | 205 | 95.60 | 5 |
| E* | *HpaII/MspI* 3.5 | *EcoR*I 3.13 | 197 | 92.95 | 11 |
| F | *HpaII/MspI* 3.5 | *EcoR*I3.10 | 180 | 94.00 | 8 |
| G | *HpaII/MspI* 3.1 | *EcoR*I 3.1 | 155 | 90.96 | 11 |
| H | *HpaII/MspI* 3.1 | *EcoR*I 3.2 | 178 | 93.00 | 1 |
| I* | *HpaII/MspI* 3.1 | *EcoR*I 3.15 | 174 | 97.70 | 9 |
| J | *HpaII/MspI* 3.1 | *EcoR*I 3.3 | 150 | 94.00 | 10 |
| K | *HpaII/MspI* 3.1 | *EcoR*I 3.13 | 149 | 94.60 | 7 |
| L | *HpaII/MspI* 3.1 | *EcoR*I3.10 | 168 | 98.00 | 4 |

Primers selected are indicated with an asterisk. The number of alleles (# of loci), percentage reproducibility of alleles (% Rep), and number of differential alleles (# diff. alleles) are displayed.
