# Supplementary material for: Common garden experiment reveals altered nutritional values and DNA methylation profiles in micropropagated three elite Ghanaian sweet potato genotypes
Source: PLoS One. 2019 Apr 26;14(4):e0208214. doi: 10.1371/journal.pone.0208214 (PMC6485893; doi:10.1371/journal.pone.0208214)
Supplement: S5 Table — ILC = Immature Leaf Colour, MLC = Mature Leaf Colour, ALVP = Abaxial Leaf Vein Pigmentation, PVC = Predominant Vine Colour, SVC = Secondary Vine Colour, PP = Petiole Pigmentation, PT = Plant Type, M = micropropagated, and F = Field-maintained plants. (DOCX) [file pone.0208214.s009.docx]

**Table S5.** Mean foliage and root quality phenotypic scores for micropropagated and field-maintained populations of three sweet potato genotypes Bohye, Ogyefo, and Otoo.

| Genotype | ILC | MLC | ALVP | PVC | SVC | PP | PT | Skin colour | Root shape | Flesh colour |
| --- | --- | --- | --- | --- | --- | --- | --- | --- | --- | --- |
| Bohye M | 2 | 2 | 7 | 3 | 6 | 3 | 5 | 5 | 2 | 7 |
| Bohye F | 2 | 2 | 7 | 3 | 6 | 3 | 5 | 5 | 2 | 7 |
| Ogyefo M | 2 | 3 | 3 | 7 | 3 | 1 | 7 | 9 | 3 | 1 |
| Ogyefo F | 2 | 3 | 3 | 7 | 3 | 1 | 7 | 9 | 3 | 1 |
| Otoo M | 2 | 2 | 8 | 4 | 6 | 4 | 7 | 2 | 8 | 6 |
| Otoo F | 2 | 2 | 8 | 4 | 6 | 4 | 7 | 2 | 8 | 6 |

M=Micropropagated plants, F=Field-maintained plants, ILC=Immature Leaf Colour, MLC=Mature Leaf Colour, ALVP=Abaxial Leaf Vein Pigmentation, PVC=Predominant Vine Colour, SVC=Secondary Vine Colour, PP=Petiole Pigmentation, PT=Plant Type, M=micropropagated, and F=Field-maintained plants.
